# Definitions and determinants of success in left bundle branch area pacing: a systematic review

**DOI:** 10.3389/fcvm.2026.1957797

**Published:** 2026-10-02

**Authors:** Jie He, Jianhui Tang, Changjiu Huang, Chuan Zhang, Xiaofen Liu, Fei Wang, Zhuoyuan Yu

**Affiliations:** 1 Department of Cardiology, Jintang Second People’s Hospital, Chengdu, Sichuan, China; 2 Department of Urology, The First Affiliated Hospital of Chongqing Medical University, Chongqing, China

**Keywords:** cardiac magnetic resonance, conduction-system capture, left bundle branch area pacing, preprocedural prediction, procedural success, septal fibrosis

## Abstract

**Background:**

Left bundle branch area pacing (LBBAP) achieves more physiological ventricular activation by advancing the lead deep into the interventricular septum and recruiting the left conduction system. Although alternative pacing is usually available when LBBAP is unsuccessful, difficult cases may undergo repeated nonproductive septal deployments before crossover, with longer procedure and fluoroscopy times and greater myocardial injury. Preprocedural prediction may therefore improve implantation and backup planning and reduce unnecessary procedural burden.

**Methods:**

PubMed and Embase were searched from inception through July 16, 2026. Two reviewers independently screened the literature. Ninety analytic full-text reports were included; preprocedural determinants formed the primary review question, while later-stage evidence was retained to contextualize endpoint definitions and clinical translation.

**Results:**

Among 90 analytic full-text reports, evidence was concentrated on capture verification, whereas only 10 evaluated preprocedural determinants. Recurrent candidate signals included left ventricular enlargement, nonclassical conduction phenotypes, and septal scar; no clinically validated threshold was identified. Septal scar was the most anatomically direct preprocedural marker of mechanical deployment difficulty, with scar location and burden linked to the intended lead path in two observational CMR cohorts. Only one multivariable prediction model was identified and it lacked external validation. Acute procedural outcomes varied by endpoint definition and clinical indication and were not directly comparable.

**Conclusion:**

Left ventricular enlargement, nonclassical conduction phenotypes, and septal scar may serve as candidate risk indicators for LBBAP procedural difficulty, although no clinically validated thresholds are currently available. When CMR is already available or clinically indicated, septal scar distribution may add information to substrate assessment and estimation of mechanical difficulty; whether dedicated CMR acquisition and CMR-informed planning improve implantation efficiency or outcomes remains untested. Routine CMR screening is unsupported. For preprocedural studies specifically targeting successful left conduction-system recruitment, confirmed capture may serve as a preferred mechanistic anchor endpoint. Mechanical deployment, indication-specific electrical correction, procedural complexity, crossover, and long-term benefit should be reported separately. Future models should incorporate operator and implant-system factors and demonstrate calibration, external validation, and clinical impact.

**Systematic Review Registration:**

https://www.crd.york.ac.uk/PROSPERO/view/CRD420261452030, PROSPERO CRD420261452030.

## 1 Introduction

Left bundle branch area pacing (LBBAP) has evolved from an experimental deep-septal pacing technique into a major form of cardiac physiologic pacing. Early studies demonstrated that a ventricular lead could be advanced deep into the interventricular septum to achieve stable electrical parameters and rapid left ventricular activation in patients with bradycardia or heart failure (1, 2). Contemporary consensus documents and reviews now describe implantation technique, clinical indications, and confirmation of conduction-system capture in considerable detail (3–7).

Although an alternative pacing strategy is usually available when LBBAP cannot be achieved, difficult procedures may involve repeated septal deployments before crossover. Failed procedures have been associated with more implantation attempts and substantially longer procedure and fluoroscopy times, while repeated lead repositioning has been associated with greater periprocedural myocardial injury (8, 9). Preprocedural prediction could therefore help identify patients likely to require a more difficult implantation, guide procedural preparation and backup planning, and reduce prolonged nonproductive attempts.

However, evidence on preprocedural prediction remains fragmented across patient characteristics, imaging findings, procedural endpoints, and definitions of success. This makes it difficult to determine which factors are clinically useful before implantation and which outcomes future prediction studies should target. The principal objective of this review was to synthesize factors available before lead advancement that were associated with acute LBBAP feasibility, failure, or procedural complexity. A secondary objective was to map success definitions across intraprocedural guidance, capture verification, and postimplant follow-up so that future prediction studies can align the predicted endpoint with the relevant mechanism and clinical decision. Procedural complexity was evaluated as a parallel outcome.

## 2 Methods

### 2.1 Search strategy and eligibility

The review followed PRISMA 2020 reporting guidance (10) and was prospectively registered in PROSPERO (CRD420261452030). PubMed and Embase.com were searched from inception through July 16, 2026, using database-specific strategies for LBBAP, LBBP, left ventricular septal pacing, and related terms. Complete search strategies, platforms, dates, and results are provided in Supplementary Table S1.

Eligible reports evaluated patient, myocardial, anatomical, procedural, diagnostic, or lead-related factors associated with LBBAP feasibility, procedural complexity, capture, postimplant response, prognosis, or durability. Descriptive case reports without an analyzable factor-outcome or diagnostic question, reviews, editorials, and unrelated pacing comparisons were excluded. Full analytic reports formed the principal synthesis. The preprocedural domain informed the primary review question; later stages were retained to clarify endpoint definition, diagnostic confirmation, clinical translation, and durability. Conference abstracts and other non-full-text reports were catalogued separately and were not used as principal evidence in the narrative synthesis.

Two reviewers independently screened titles and abstracts and assessed full-text reports, with disagreements resolved by discussion and consensus. No language restriction was applied at the search stage. Reference lists of included reports and relevant reviews were checked for additional eligible reports; a formal forward-citation search was not conducted. The report-selection process is shown in Figure 1.

**Figure 1:**
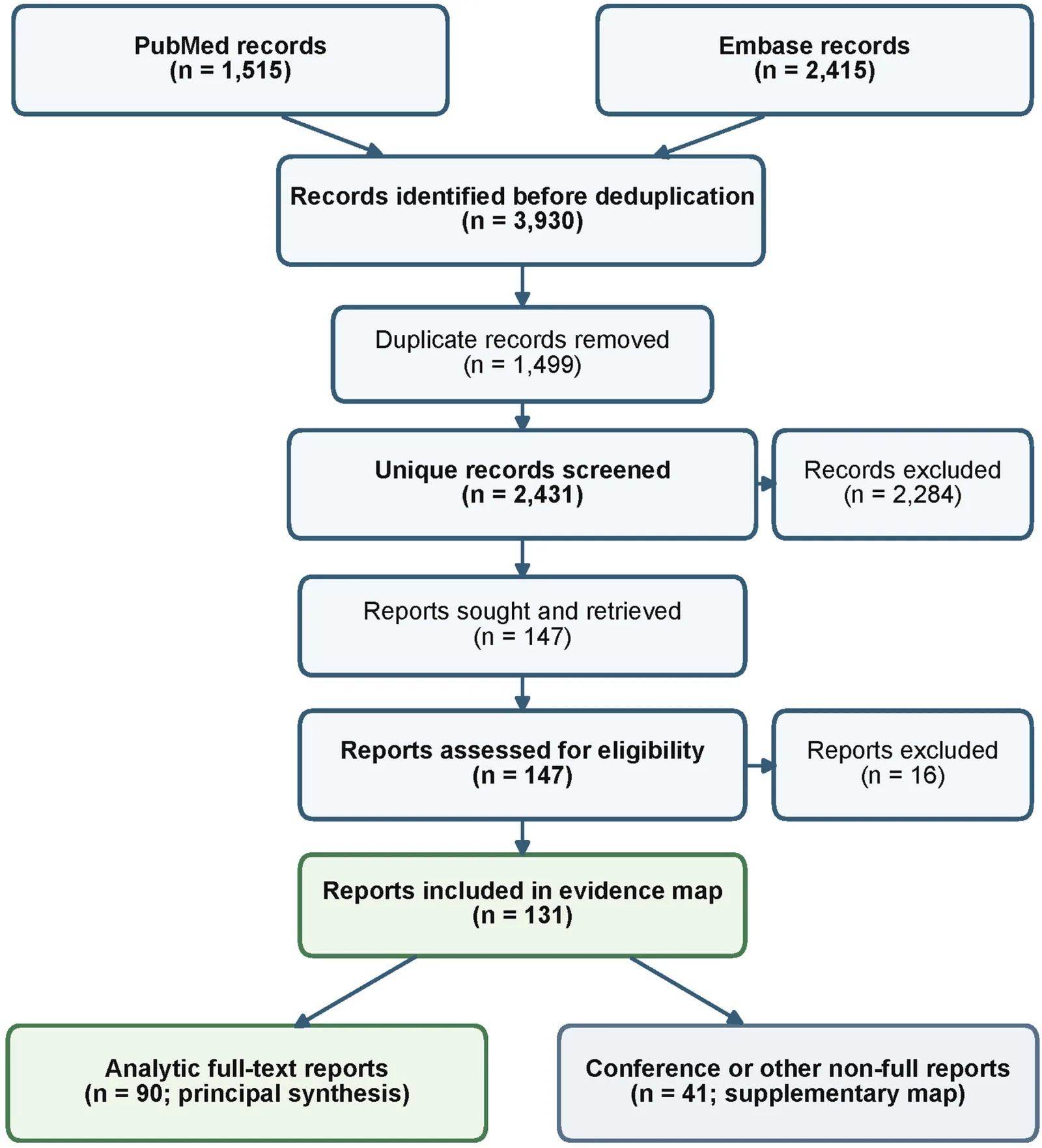
PRISMA-style report flow. All 147 sought reports were retrieved and assessed. The review included 90 analytic full-text reports in the principal synthesis and catalogued 41 conference abstracts and other non-full-text reports separately.

### 2.2 Analytic framework and success hierarchy

Evidence was organized into four clinical stages: preprocedural determinants, intraprocedural guidance, capture verification, and postimplant translation or durability. The final five-level hierarchy was not prespecified as a formal classification used for study selection; it was developed during narrative synthesis to map the outcome domains represented in the protocol and retrieved literature. The hierarchy comprises mechanical deployment, broad LBBAP implantation, confirmed left conduction-system capture, acute electrical correction, and durable physiological or clinical benefit. It is an author-derived proposed framework and has not undergone formal consensus development or external validation. Procedural complexity was retained as a parallel outcome axis. The framework is summarized in Table 1.

**Table 1:** Evidence synthesis framework for LBBAP outcomes.

Stage	Clinical question	Typical evidence	Role in the synthesis
Preprocedural determinants	Which mechanism of difficulty or failure is plausible before implantation?	Clinical history, indication, ECG, echocardiography, CMR/CT, baseline models	Identifies difficult-substrate phenotypes and factors that may inform further substrate characterization or procedural preparation.
Intraprocedural guidance	Does the current site and trajectory remain promising and safe?	Paced morphology, activation timing, current of injury, depth, fixation beats, septography, echocardiography	Manages residual uncertainty after the procedure has begun and helps determine whether additional attempts remain productive or crossover is appropriate.
Capture verification	Has true conduction-system capture been achieved?	V6 RWPT/LVAT, V6-V1 sequence, output transition, morphology, intracardiac signals, ECGI	Defines one specific procedural endpoint using convergent physiological evidence.
Postimplant translation and durability	Did acute technical success produce benefit and persist?	Scar burden, capture certainty, electrical response, remodeling, lead position, thresholds, remote electrograms	Validates the clinical meaning and durability of earlier success levels.

### 2.3 Data extraction and synthesis

Data were extracted using a structured full-text extraction form. One reviewer performed the initial extraction, and a second reviewer verified the extracted information against the source reports, with discrepancies resolved by discussion and consensus. Extracted items included study design and setting, population and indication, sample size and analytic unit, candidate factor or index test, outcome or reference standard, principal results, statistical approach, follow-up, validation status, lead and delivery system, operator or center characteristics when reported, and potential report overlap. Potentially overlapping reports were identified using study center, investigators, recruitment period, population, and sample size. When multiple reports represented the same cohort and the same factor-outcome comparison, the most complete or appropriately adjusted report was prioritized for inference, while distinct analyses addressing different questions were retained in the evidence map. Study characteristics and principal findings are reported in Supplementary Table S2, and conference abstracts and other non-full-text reports are catalogued in Supplementary Table S7.

Owing to substantial heterogeneity in populations, endpoints, and study methods, evidence was synthesized narratively. For the descriptive acute-success analysis, study–endpoint pairs were eligible when at least one factor was available before lead advancement, the outcome was an acute patient-level procedural endpoint, and an unambiguous numerator and denominator were available. Distinct success definitions were analyzed separately, and Wilson 95% confidence intervals were calculated without producing a pooled estimate. Detailed eligibility rules and source data are provided in Supplementary Methods and Supplementary Table S8.

### 2.4 Risk-of-bias assessment

Two reviewers independently assessed risk of bias, with disagreements resolved by discussion and consensus. QUIPS was used for prognostic-factor studies, QUADAS-2 for diagnostic and capture-verification studies, PROBAST for the multivariable prediction model, and ROBINS-I for nonrandomized comparative studies. Study-level and domain-level judgments are reported in Supplementary Tables S3–S6.

## 3 Results

### 3.1 Study flow and distribution of the evidence

The searches identified 3,930 records. After removal of 1,499 duplicates, 2,431 unique records were screened and 147 reports were retrieved for full assessment. Sixteen were excluded. The principal synthesis comprised 90 analytic full-text reports, while 41 conference abstracts and other non-full-text reports were catalogued separately in Supplementary Table S7.

The evidence was unevenly distributed across the four decision stages. Ten reports addressed preprocedural determinants, 13 intraprocedural guidance, 41 capture verification, and 26 postimplant translation or durability. By study question, 51 were predictor or prognostic-factor studies, 30 diagnostic or capture-verification studies, eight nonrandomized comparative studies, and one multivariable prediction-model study. The evidence base was therefore strongly weighted toward capture verification, with comparatively limited evidence available to guide prediction before lead advancement. The distribution by decision stage and study question is shown in Figure 2 and Table 2.

**Figure 2:**
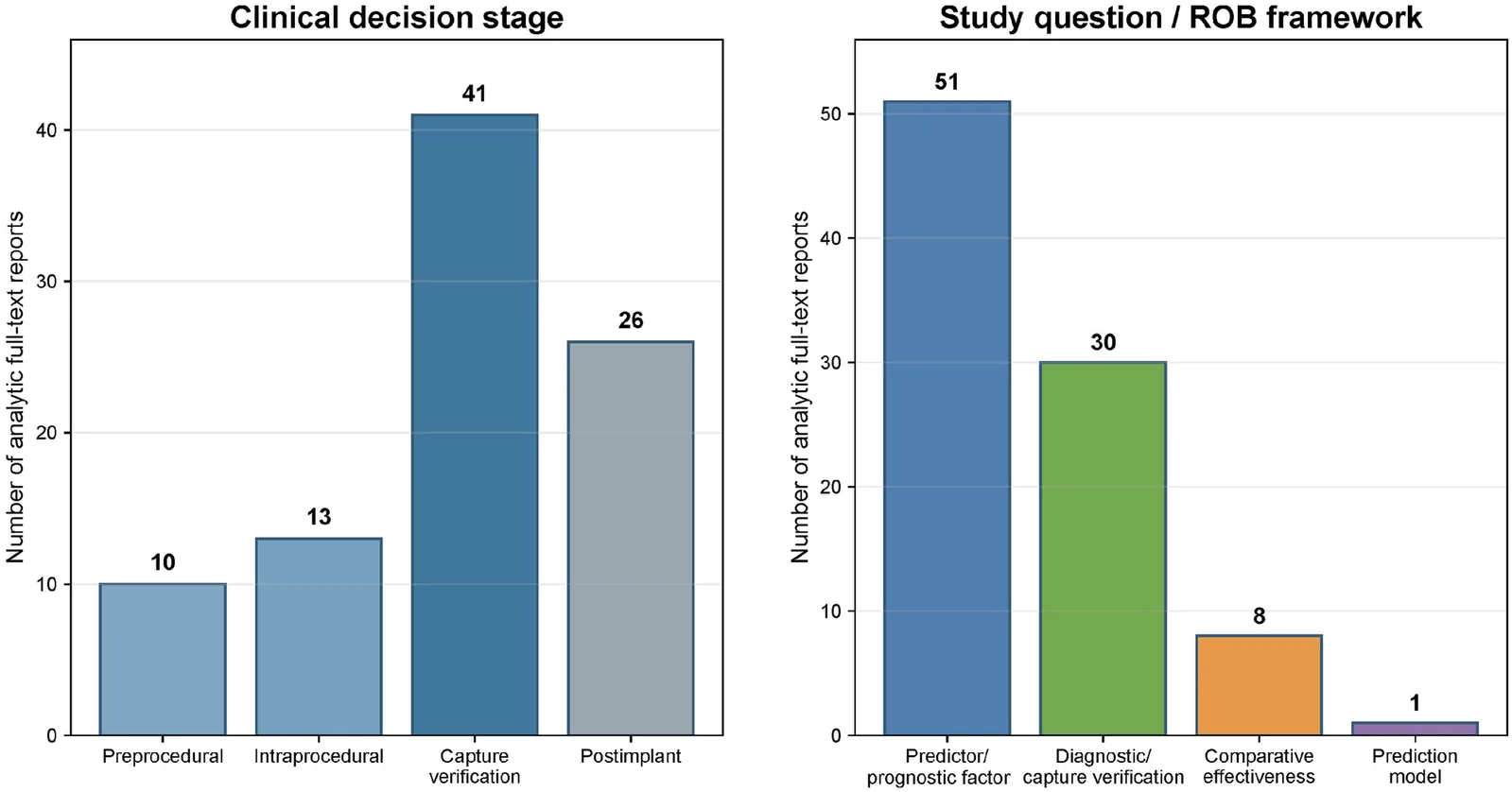
Distribution of the 90 analytic full-text reports by clinical decision stage and study question. Counts are report-level classifications and should not be interpreted as independent cohorts or pooled effect strength.

**Table 2:** Distribution and risk of bias of the 90 analytic full-text reports.

Study question/tool	Reports	Stage: pre/intra/capture/post	Final ROB distribution
Predictor/prognostic factor (QUIPS)	51	9/7/14/21	Moderate 41; high 10
Diagnostic/capture verification (QUADAS-2)	30	0/5/24/1	Unclear 16; high 14
Nonrandomized comparison (ROBINS-I)	8	0/1/3/4	Moderate 5; serious 3
Multivariable prediction model (PROBAST)	1	1/0/0/0	High 1
Total	90	10/13/41/26	Categories interpreted within tools

### 3.2 Reported acute success by endpoint and population

Among studies evaluating factors available before lead advancement, mechanical deployment success ranged from 64.0% to 85.7% (11, 12), broad study-defined LBBAP implantation from 86.7% to 95.8% (8, 13, 14), and strict LBBP or confirmed capture from 66.4% to 86.9% (8, 15–17). Acute electrical correction was achieved in 75.3% of the selected bundle-branch-block cohort reported by Jiang et al. (18). These strata represent different clinical populations and non-equivalent endpoints and should not be interpreted as estimates of a single standardized success probability. They were therefore not pooled or compared as one outcome. Figure 3 displays the eligible study-endpoint pairs by endpoint definition and clinical indication. Table 3 summarizes the main patient-level studies of acute feasibility, failure, or procedural complexity, and Table 4 presents the proposed reporting framework.

**Figure 3:**
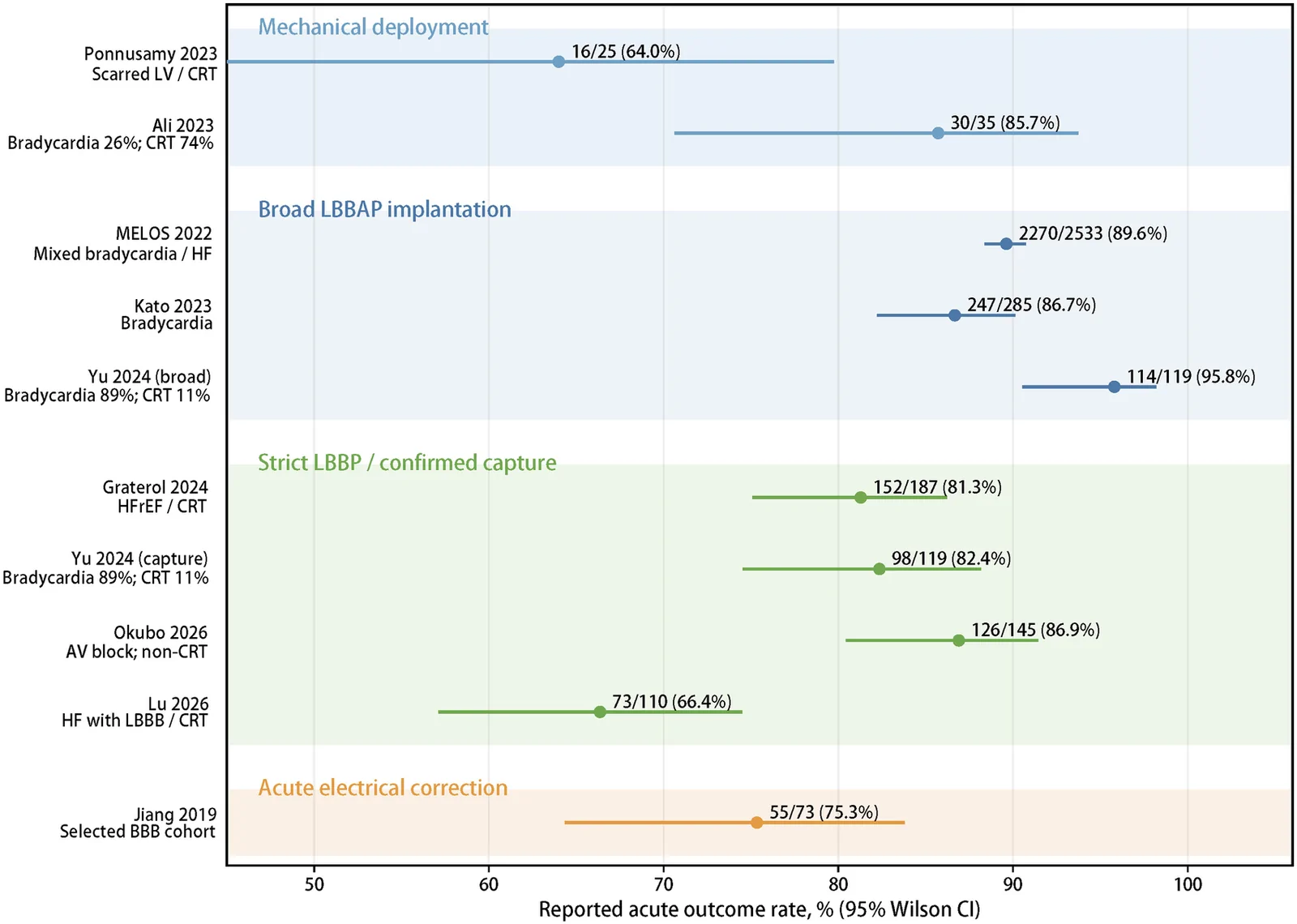
Reported acute procedural outcomes across studies evaluating factors available before lead advancement. Studies are stratified by endpoint definition, and clinical indication is shown in each study label. Eligibility required a patient-level procedural success endpoint and an unambiguous numerator and denominator. Distinct non-equivalent endpoints from the same report were displayed separately. Wilson 95% confidence intervals are shown. The proportions describe different clinical populations and operational definitions and were not pooled or interpreted as one standardized outcome. Eligibility criteria and source data are provided in Supplementary Methods and Supplementary Table S8.

**Table 3:** Main studies of acute LBBAP feasibility, failure, or procedural complexity.

Study/population/indication/ROB	Lead/operator/setting	Endpoint/failures	Predictor/adjusted or principal result
MELOS 2022; mixed bradycardia/HF; *n* = 2,533 ROB: Moderate	Center-specific systems; lead type not separated in predictor analysis; 14 European centers; learning curve assessed	Study-defined broad LBBAP; 263 failures	LVEDD: adjusted OR 1.53 per 10 mm
Kato 2023; bradycardia; *n* = 285 ROB: Moderate	SelectSecure 3830 lumenless lead; delivery tools varied by center; multicenter; operator experience not modeled	Study-defined broad LBBAP; 38 failures	IVCD: adjusted OR 8.16 (95% CI 2.32–28.75)
Ponnusamy 2023; scarred LV/CRT; *n* = 25 ROB: High	SelectSecure 3830 + C315 sheath; single center; operator experience not reported	Mechanical septal penetration; 9 failures	Transmural LGE in LBBP zone: 9/9 failures vs 0/16 successes
Ali 2023; bradycardia 26%, CRT 74%; *n* = 35 ROB: High	SelectSecure 3830 lumenless lead; transseptal approach; prospective single-operator cohort	Mechanical LV-septal deployment; 5 failures	Median septal scar: 54% in failures vs 8% in successes (*p* < 0.001)
Graterol 2024; HFrEF/CRT; *n* = 187 ROB: Moderate	SelectSecure 3830 lumenless lead + dedicated delivery sheath; single center; operator experience not modeled	Study-defined LBBP; 35 failures	Non-LBBB: OR 3.07; LVEDD: OR 1.31 per 5 mm
Yu 2024; bradycardia 89%, CRT 11%; *n* = 119 ROB: Moderate	Solia S60 stylet-driven lead + Selectra 3D sheaths; 3 centers; operator experience described in source	Broad LBBAP: 5 failures; confirmed capture: 21 failures	IVCD and RA size predicted more attempts; >3 attempts lowered success
Okubo 2026; AV block, non-CRT; *n* = 145 ROB: High	SelectSecure 3830 + C315 His sheath; single high-volume center; operators beyond early learning phase	Study-defined confirmed capture; 19 failures	NIVCD: OR 4.34; model AUC 0.84; bootstrap AUC 0.85
Lu 2026; HF with LBBB/CRT; *n* = 110 ROB: Moderate	Lead/delivery system not reported in extracted characteristics; single center; operator experience not reported	Study-defined LBBP; 37 failures	Concordant LBBB: OR 3.90 for procedural success

**Table 4:** Author-derived proposed framework for reporting LBBAP outcomes.

Level	Endpoint term	Minimum meaning	Scope limits	Recommended research use
1	Mechanical deployment success	Lead advanced to the intended deep-septal or LV-side position with acceptable acute lead parameters.	Conduction-system capture and electrical correction require separate assessment.	Mechanical feasibility and scar- or path-related failure prediction.
2	Broad LBBAP implantation success	Final implant meets the study-defined LBBAP endpoint; reports state whether LV septal pacing counts as success.	Confirmed left conduction-system capture requires additional physiological evidence.	Service-level feasibility with an explicit operational definition.
3	Confirmed left conduction-system capture	Prespecified physiological evidence supports recruitment of the left conduction system.	Electrical correction and later clinical benefit require separate assessment.	Preferred mechanistic anchor endpoint for preprocedural studies specifically targeting left conduction-system recruitment; also supports capture-specific diagnostic validation.
4	Acute electrical correction	Prespecified indication-specific electrical target is achieved during the index procedure.	Deployment and capture are recorded separately.	Acute therapeutic translation, especially in CRT or bundle-branch-block populations.
5	Durable physiological or clinical success	Capture persists and prespecified electrical, imaging, functional, or clinical benefit is documented over time.	Acute technical completion alone cannot establish this level.	Long-term effectiveness, durability, and prognosis.
Parallel axis	Procedural complexity	Deployment attempts, procedure and fluoroscopy time, myocardial injury, complications, crossover, and strategy change.	Reported independently from the final success level.	Burden prediction, stopping rules, and clinical-impact evaluation.

### 3.3 Preprocedural determinants of initial feasibility

#### 3.3.1 Routine ECG, echocardiographic, and clinical variables

Across preprocedural studies, marked left ventricular enlargement and nonclassical conduction phenotypes [defined in the source studies as non-LBBB morphology, intraventricular conduction delay (IVCD), or nonspecific intraventricular conduction delay (NIVCD)] recurred in cohorts with lower procedural success or greater complexity (8, 13–17). MELOS reported an adjusted association between larger LV end-diastolic diameter and unsuccessful implantation, while Graterol et al. observed independent associations of non-LBBB morphology and greater LV size with failed LBBP in HFrEF candidates (13, 15). Kato et al. and Okubo et al. identified IVCD or NIVCD among the strongest baseline signals, with different success definitions across studies (14, 16). Yu et al. further linked IVCD and right atrial enlargement with a greater number of implantation attempts (8). No clinically validated threshold was identified for these candidate predictors.

The candidate variables were modeled using different units, thresholds, covariates, and endpoints. The only formal multivariable prediction model included 19 unsuccessful cases, reported calibration assessment, and underwent internal bootstrap validation only; external validation, decision-curve analysis, and prospective clinical-impact testing were unavailable. The model was judged at high risk of bias (16).

Lead and delivery systems varied across the main acute predictor studies. Most lumenless-lead cohorts used the SelectSecure 3830 system, while Yu et al. evaluated the Solia S60 stylet-driven lead with Selectra 3D sheaths (8). Four of eight main studies described operator structure or experience sufficiently to characterize the procedural environment; only MELOS explicitly evaluated a learning curve. Operator experience and center volume were not handled consistently in adjusted predictor analyses. Table 3 summarizes the available study-level details.

#### 3.3.2 CMR-defined scar and the septal substrate

Septal scar was the most anatomically direct preprocedural marker of mechanical deployment difficulty. In two observational cohorts totaling 60 patients and 14 deployment failures, scar burden or transmural fibrosis was localized to the intended lead path and associated with failure of septal penetration or target attainment (11, 12). In a selected scarred-LV cohort, all nine procedural failures and none of the 16 successes had transmural late gadolinium enhancement in the intended LBBP zone (11). In a prospective CMR cohort, median septal scar burden was 54% among five deployment failures and 8% among 30 successes (12). Both studies were judged at high risk of bias; no threshold or effect estimate can therefore be considered clinically validated.

CMR-derived global, septal, and lead-site scar was also associated with remodeling and prognosis after LBBAP-CRT (19–22), supporting the broader relevance of myocardial substrate to downstream response. No study evaluated whether access to CMR findings changed target selection, equipment preparation, deployment attempts, procedure or fluoroscopy time, crossover decisions, or clinical outcomes.

### 3.4 Intraprocedural guidance signals

V6 R-wave peak time (RWPT) measured during right ventricular septal pacing can provide an early estimate of the likelihood that a candidate site will achieve conduction-system recruitment. Cai et al. reported that a longer V6 RWPT was associated with a lower likelihood of successful LBBAP: for each 10-ms prolongation, the adjusted odds of success decreased by approximately 58% (OR, 0.42), with good discrimination between successful and unsuccessful procedures (AUC, 0.888) (23). Paced QRS morphology and quantitative parameters of lead position were also associated with the initial septal site and subsequent acute electrical correction or implantation success (18, 24, 25).

Other signals described lead advancement, contact, fixation, and safety. Current-of-injury amplitude and tip:ring relationships, fixation beats, transseptal transition patterns, lead depth, septography, CT-informed anatomy, and intraprocedural echocardiography were evaluated across patients, attempted sites, deployments, ECGs, and leads (26–34). Differences in population, analytic unit, and outcome precluded a common effect estimate.

### 3.5 Capture-verification criteria

Capture verification was the largest domain. Fixed V6 RWPT or LVAT thresholds were simple and immediately available but varied with baseline QRS pattern, cardiomyopathy, pacing location, and capture selectivity (25, 35–39). Individualized activation-time criteria referenced His-bundle or RV septal pacing, while V6-V1 interpeak criteria incorporated the relative sequence of left and right ventricular activation (40, 41). Output-dependent transitions, morphology, intracardiac signals, continuous pacing-and-recording, ECG imaging, frequency-domain measures and composite algorithms provided complementary physiological evidence (42–51).

No single criterion was consistently validated across different substrates, pacing locations, and clinical settings. The criterion families differed in availability, dependence on fixed thresholds, and the type of physiological evidence provided. The major families are compared in Table 5.

**Table 5:** Interpretation of major capture-verification criterion families.

Criterion family	Physiological idea	Main advantage	Main vulnerability	Best current interpretation
Fixed V6 RWPT/LVAT	Rapid lateral LV activation suggests conduction-system recruitment.	Simple and immediately available.	Baseline QRS, substrate, and pacing-site dependence; threshold transferability is limited.	Initial screen rather than sole proof in abnormal substrate.
Individualized activation-time change	Patient-specific reference pacing defines the expected timing improvement.	Accounts for between-patient conduction variation.	Requires an appropriate reference and same-patient measurements.	Useful when fixed cutoffs are unreliable.
V6-V1 interpeak interval	Relative left-right activation sequence reflects conduction-system engagement.	Captures sequence rather than one absolute latency.	Threshold-derived and tested in selected cohorts.	Complement to timing and morphology.
Output-dependent transition	Capture type changes with output when conduction tissue is recruited or lost.	Strong physiological evidence when observed.	Transitions may be absent or operator dependent.	High-value confirmatory sign.
Morphology/composite criteria	Multiple ECG features jointly support capture classification.	Practical and integrative.	Weights and thresholds are often internally derived.	Structured adjudication with other signs.
ECGI/frequency-domain/advanced signals	Activation maps or spectral features provide broader electrical characterization.	Mechanistically informative and useful for validating simpler criteria.	Limited availability and external validation.	Research validation and selected difficult cases.

### 3.6 Postimplant translation and durability

Baseline substrate, including LBBB phenotype, LV size, and scar burden, was associated with reverse remodeling and prognosis after LBBAP-CRT (19–22, 52). Confirmed versus unconfirmed conduction-system capture, paced morphology, activation-time reduction, and other electrophysiological features were associated with electrical response, remodeling, or clinical outcomes (53–57). Additional studies used repolarization metrics and artificial intelligence-enabled ECG to characterize postimplant response (58, 59).

A smaller set of studies addressed the durability of capture or monitoring of late change. Lead-tip distance from the LV cavity, threshold behavior, late capture loss, and remote electrogram analysis were evaluated as potential indicators of long-term stability (60–63). Most studies were observational, internally derived, and potentially affected by patient selection, substrate, and uncertainty in the original capture classification.

### 3.7 Procedural complexity as a parallel outcome

A small number of studies directly evaluated procedural burden. Failed procedures were associated with more deployment attempts and substantially longer procedure and fluoroscopy times, while repeated lead repositioning was independently associated with marked periprocedural myocardial injury (8, 9). Transseptal-route complications were also reported, although current evidence does not establish a dose-response relationship between the number of attempts and discrete complications (13).

### 3.8 Risk of bias

Risk of bias was generally moderate to high across the included literature. Common concerns included selected cohorts, small event counts, inconsistent analytic units, internally derived thresholds, incomplete control of confounding, imperfect reference standards, lack of blinding, and limited external validation. These concerns constrained inference: LV enlargement and conduction phenotypes were interpreted as recurrent candidate associations, the CMR signal as anatomically direct but based on sparse observational evidence, and the single formal prediction model as exploratory pending external validation. Tool-specific judgments are summarized in Table 2 and reported in detail in Supplementary Tables S3-S6.

## 4 Discussion

### 4.1 An anchor endpoint for prediction studies

The central implication of this review is that preprocedural prediction cannot mature until it is tied to a clearly defined procedural endpoint. For studies specifically designed to predict successful left conduction-system recruitment, we propose confirmed capture at the end of the index implantation, assessed among all patients in whom LBBAP was attempted using prespecified physiological criteria, as a preferred mechanistic anchor endpoint. This endpoint is physiologically specific, ascertainable during the index procedure, and less influenced by downstream disease severity and subsequent treatment than remodeling or clinical events. Other clinical questions may appropriately prioritize mechanical deployment, indication-specific electrical correction, durable capture, or clinical benefit.

Mechanical deployment should be reported when the question concerns septal penetrability or target access; indication-specific electrical correction when acute therapeutic effect is the question; and durable capture or clinical benefit when long-term translation is the question. The five-level framework records the outcome achieved, while the procedural-complexit*y* axis records the burden required to reach it. Crossover, complications, and strategy change remain separate outcomes. The framework is an author-derived synthesis tool; prospective consensus and validation are required before it can be adopted as a reporting standard.

### 4.2 Prediction must be mechanism-specific and transportable

The candidate predictors identified in this review are unlikely to act at the same point in the procedure. Septal fibrosis may primarily limit mechanical penetration or target attainment (11, 12); chamber enlargement may alter sheath support and access; diffuse conduction disease may affect capture or electrical correction (8, 13–17); and the lead-tissue interface may influence durability. Combining these mechanisms into a single overall failure label may produce a model that discriminates risk without identifying a clinically actionable response.

Future studies should prespecify mechanical deployment failure, failure to confirm left conduction-system capture, failure of indication-specific electrical correction, excessive procedural complexity, and late loss of capture as distinct outcomes. The selected endpoint should match the hypothesized mechanism and connect to a plausible procedural action.

LBBAP outcome is influenced by patient substrate, operator experience, center volume, lead and sheath systems, imaging and mapping resources, and the endpoint being pursued. Current studies reported these procedural-environment variables inconsistently, limiting transportability across centers and technologies. Future models should record intrinsic patient factors and procedural-environment factors and evaluate discrimination, calibration, external validation, and decision-curve performance. Prospective clinical-impact studies should then test whether model-guided planning changes operator assignment, equipment preparation, imaging strategy, crossover timing, deployment attempts, procedure and fluoroscopy time, or myocardial injury.

### 4.3 CMR as a candidate decision tool

Among the preprocedural signals identified in this review, septal scar is distinctive because CMR can localize scar burden, transmurality, and distribution along the intended lead path. This spatial information is not directly supplied by routine ECG or echocardiographic variables and provides a mechanistic link to septal penetrability and target attainment. When CMR is already available or clinically indicated, scar distribution may therefore add relevant information to preprocedural substrate assessment and estimation of mechanical difficulty. What remains untested is the incremental value of obtaining CMR specifically for LBBAP planning and using those findings to alter target selection, equipment preparation, or crossover strategy (11, 12, 19–22).

Marked ventricular remodeling, nonclassical conduction disease, ischemic or structural myocardial disease, known septal injury, or prior imaging abnormalities may increase the pretest likelihood of septal fibrosis and may make existing or otherwise indicated CMR particularly informative. These characteristics are not validated LBBAP-specific indications for CMR and do not constitute a formal selection rule. Prospective impact studies should test whether dedicated CMR acquisition and CMR-informed planning change target selection, deployment attempts, procedure and fluoroscopy time, myocardial injury, crossover, or final pacing outcome. Routine CMR screening and exclusion from LBBAP on the basis of CMR findings alone remain unsupported.

### 4.4 Strengths and limitations

Strengths of this review include prospective registration, dual-database searching, independent screening, structured extraction with second-reviewer verification, design-specific risk-of-bias assessment, and a stage-based synthesis of 90 analytic full-text reports.

Limitations reflect both the literature and the review design. Evidence was heterogeneous in population, operator experience, lead system, analytic unit, outcome definition, and reference standard, and several reports came from overlapping programs. Many studies were small, single-center, internally derived, or lacked external validation. The five-level reporting framework is an author-derived interpretive output and remains unvalidated. Evidence linking septal scar to deployment failure came from two small observational cohorts, and no study evaluated the clinical impact of CMR-informed procedural planning. Operator experience and center volume were incompletely reported and inconsistently modeled across predictor studies.

## 5 Conclusions

Current evidence identifies recurrent candidate signals for LBBAP procedural difficulty, including left ventricular enlargement and nonclassical conduction phenotypes, although no clinically validated thresholds are available. Septal scar is the most anatomically direct preprocedural substrate marker of mechanical deployment difficulty identified in the current evidence. When CMR is already available or clinically indicated, scar distribution may contribute to procedural risk assessment; whether dedicated CMR acquisition and CMR-informed planning improve implantation efficiency or outcomes remains unknown and should be tested prospectively. Routine CMR screening or exclusion from LBBAP on the basis of CMR alone is unsupported. For preprocedural studies specifically targeting successful left conduction-system recruitment, confirmed capture may serve as a preferred mechanistic anchor endpoint. Mechanical deployment, indication-specific electrical correction, procedural complexity, crossover, and durable benefit should be reported separately. Future models should account for operator and implant-system factors and demonstrate calibration, external validation, decision-curve performance, and prospective clinical impact.

## Data Availability

The original contributions presented in the study are included in the article/Supplementary Material, further inquiries can be directed to the corresponding author.
